# A Rare Oral Metastatic Lesion as the Initial Diagnosis of Small Cell Lung Cancer: Case Report and Systematic Review

**DOI:** 10.3390/jcm15103772

**Published:** 2026-05-14

**Authors:** Gioele Gioco, Iacopo Cioccoloni, Cosimo Rupe, Francesca Beccari, Domenico De Falco, Mariantonietta Di Salvatore, Guido Rindi, Carlo Lajolo

**Affiliations:** 1 Head & Neck Department, Fondazione Policlinico Universitario A. Gemelli IRCCS, Università Cattolica del Sacro Cuore, 00168 Rome, Italy; gioele.gioco@unicatt.it (G.G.); dottorcioccoloni@gmail.com (I.C.); francescabeccari@icloud.com (F.B.); carlo.lajolo@unicatt.it (C.L.); 2 Interdisciplinary Department of Medicine, University of Bari, 70121 Bari, Italy; defalcodomenico@ymail.com; 3 Comprehensive Cancer Center, Medical Oncology Department, Fondazione Policlinico Universitario A. Gemelli IRCCS, 00168 Rome, Italy; mariantonietta.disalvatore@policlinicogemelli.it; 4 Section of Anatomic Pathology, Department of Life Sciences and Public Health, Università Cattolica del Sacro Cuore, 00168 Rome, Italy; guido.rindi@unicatt.it; 5 Anatomic Pathology Unit, Department of Laboratory Science and Hematology, ENETS Center of Excellence, Fondazione Policlinico Universitario Agostino Gemelli IRCCS, Università Cattolica del Sacro Cuore, 00168 Rome, Italy

**Keywords:** oral metastasis, small cell lung cancer, systematic review, case report, gingival metastasis

## Abstract

**Objectives**: This study aims to describe a rare case of oral metastasis from small-cell neuroendocrine carcinoma originating from the lung and to perform a systematic review of the cases reported in the literature. **Methods**: We present the case of a patient with oral metastasis from small-cell neuroendocrine carcinoma of the lung. The patient presented at the clinical examination with severe pain in the attached gingiva in the 3.5 region, extracted one month earlier, despite prolonged antibiotic therapy. A systematic review of the literature was conducted using the PubMed, Scopus and Web of Science databases, in accordance with the PRISMA 2020 guidelines for systematic reviews. **Results**: A biopsy of the affected area was performed. The histological and immunohistochemical analysis revealed a fragment with features compatible with a secondary lesion with probable origin from the lung. In addition, a review of the existing English-language literature was carried out and revealed a total of six cases of oral metastasis. **Conclusions**: Oral metastases from small-cell neuroendocrine carcinoma are rare but have been reported in the literature. The gold standard for diagnosis remains histological examination.

## 1. Introduction

Oral metastases are approximately 1–3% of all malignant lesions of the oral cavity [1,2,3], of which 23% represent the first diagnosis of tumors with previously unknown primary sites [4]. The main neoplasms associated with oral metastases are hepatic, pulmonary, renal, and prostatic carcinomas [5]. However, there are also other types of neoplasms that may metastasize, although with a lower incidence, such as metastases from colorectal adenocarcinoma or thymomas [6,7]. Malignant lung lesions represent the second most common malignancy in female patients and the most frequent in male patients; in 2020, approximately 2.2 million new cases and 1.8 million deaths were reported [8,9]. Smoking and tobacco consumption habits are strongly associated with the development of lung cancer. Nicotine and its derivatives, present in tobacco and smoke, contribute to promoting the expression of oncogenic proteins that lead to cancer development [10].

Lung neoplasms can be classified as: malignant epithelial carcinomas, malignant neuroendocrine tumors, malignant mesenchymal tumors typical of the lung, malignant hematolymphopoietic tumors, and malignant tumors of ectopic tissues. Lesions histologically belonging to the group of small-cell tumors fall within neuroendocrine neoplasms [11], which are typically diagnosed at advanced stages and are characterized by poor prognosis [12,13].

Small-cell neuroendocrine carcinomas show rapid progression with early metastasis and extensive systemic dissemination [14]. The survival rate is approximately 7% at 5 years from oncological diagnosis, despite initial sensitivity to the cytotoxic drugs used in oncological treatment [13,14]. Metastases from small-cell lung carcinoma spread mainly through the bloodstream and affect distant organs such as the liver, bones, brain, lymph nodes, and more rarely the oral cavity [15]. Due to their rarity, they are not promptly diagnosed and are often misdiagnosed as other oral lesions, including benign ones, further compromising the prognosis of affected patients [16].

This study aims to describe a rare case of oral metastasis from small-cell neuroendocrine carcinoma in a 62-year-old female patient, presenting as the first clinical sign at the level of the keratinized gingiva. In addition, a systematic review of oral metastasis from small-cell neuroendocrine carcinoma reported in the literature was conducted.

## 2. Materials and Methods

### 2.1. Case Report

A 62-year-old female patient was referred to the Oral Medicine Department for severe pain in the third quadrant, exacerbated by mastication and palpation, persisting for approximately 45 days prior to the visit. Her medical history was unremarkable, and she reported no ongoing medications. The patient had a long history of smoking (approximately 20 cigarettes/day since adolescence).

Dental history revealed previous endodontic treatment of tooth 3.5 (positive to percussion) without success, followed by extraction due to persistent symptoms. After the minor surgical procedure, the specialist prescribed broad-spectrum antibiotic therapy based on Amoxicillin + Clavulanic Acid twice daily for 21 days, and Rifampicin twice daily for 4 days, without any clinical improvement.

**Figure 1:**
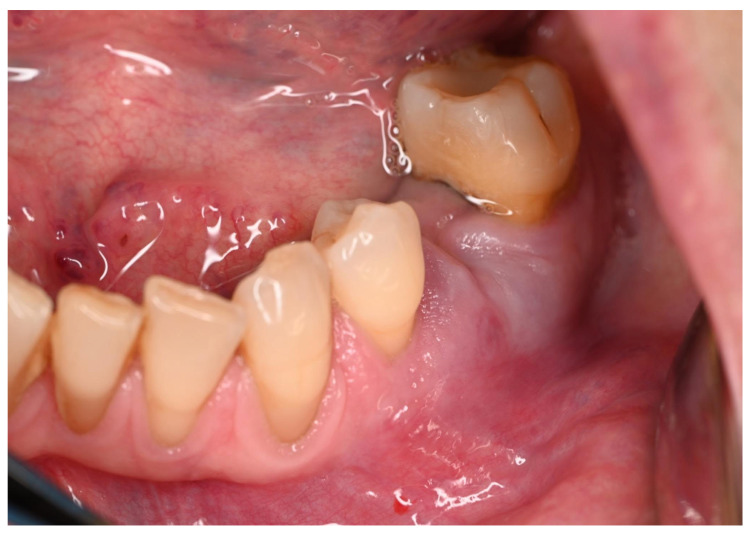
Clinical presentation of the oral lesion.

**Figure 2:**
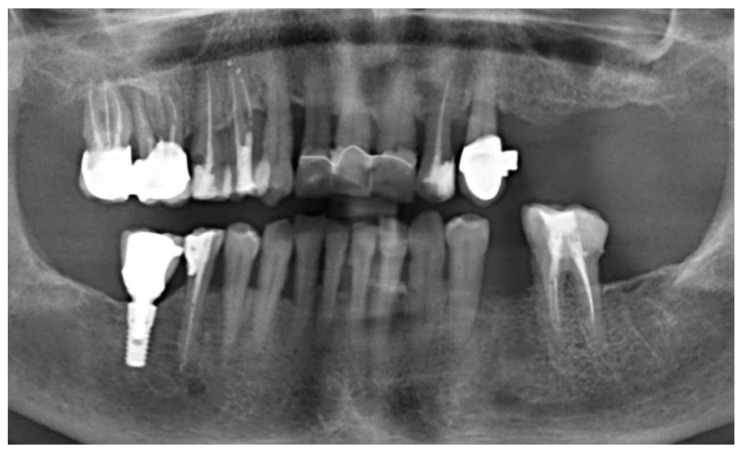
Orthopantomogram X-ray examination of the patient.

**Figure 3:**
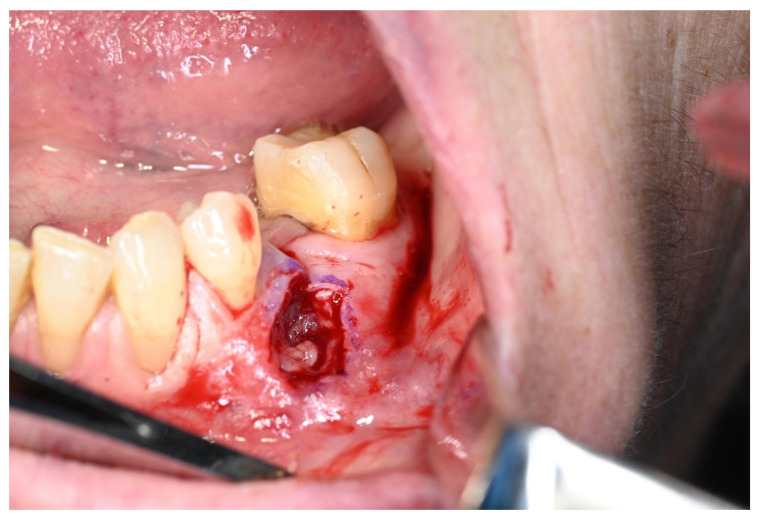
Biopsy sampling area.

**Figure 4:**
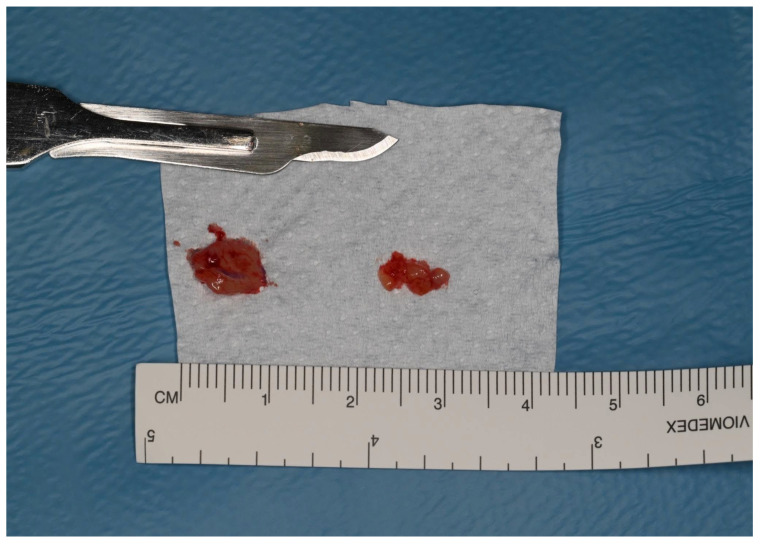
Sampling of the collected samples.

Clinical examination revealed a slightly, swollen, erythematous lesion at the extracted site, painful on palpation, soft in consistency, and fixed to the underlying tissues (Figure 1); clinical features were compatible with benign lesions such as pyogenic granuloma, peripheral giant cell granuloma, and fibrous epulis. Teeth 3.6 and 3.4 adjacent to the area of interest showed mild mobility (grade 1) and responded positively to cold vitality tests. Orthopantomography revealed a post-extraction socket at 3.5 site undergoing bone remodeling. No other evident radiographic signs were noted (Figure 2). An incisional biopsy was performed and two tissue samples were collected: (a) including epithelium and connective tissue (superficial); and (b) including connective and muscle tissue (deep) (Figure 3 and Figure 4). The area was then sutured using 4.0 PGA suture and the patient was discharged with antibiotic therapy based on Levofloxacin 500 mg twice daily for 5 days.

**Figure 5:**
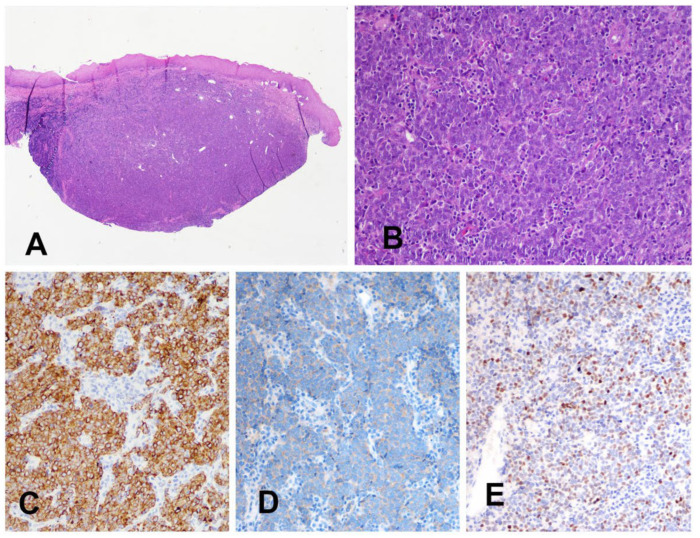
Histopathological and immunohistochemical findings of the oral lesion. Photomicrograph of the lesion. The discrete bulging of the oral mucosa with relatively expansive margins (**A**) is composed by poorly differentiated epithelioid cancer cells (**B**) positive for the cytokeratin cocktail AE1 AE3 (**C**) synaptophysin (**D**) and INSM1 (**E**). Hematoxylin and Eosin (**A**,**B**); immunoperoxidase (**C**,**D**); original magnification 20× (**A**), 200× (**B**–**E**).

Histological examination identified a normally structured oral mucosa infiltrated by an epithelioid neoplasm with poorly differentiated neuroendocrine morphology of epithelial small-cell lineage. Immunohistochemical analysis yielded positive for AE1/AE3, INSM1, synaptophysin, and INI-1, with a high proliferative index (Ki-67 ≈ 90%). The results of the immunohistochemistry are reported in Table 1. Therefore, a diagnosis of oral metastasis of unknown primary tumor of small-cell neuroendocrine carcinoma was established (Figure 5).

**Figure 6:**
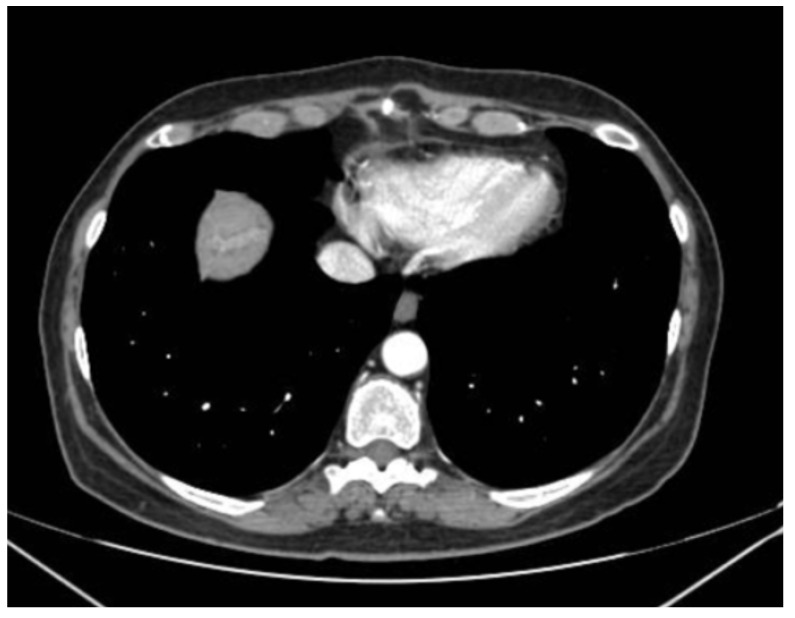
Subsolid mass in the posterior segment of the right upper lobe.

Whole-body PET-CT and MRI revealed a subsolid mass in the posterior segment of the right upper lobe, characterized by irregular and spiculated margins, pleural traction strands, a predominantly ground-glass component, and associated bronchiectatic changes. In the right lower lobe (RLL), an additional nodular lesion with irregular margins was identified (Figure 6).

According to the TNM classification, both pulmonary findings corresponded to stage III disease (T1: lesion > 7 mm; N3: involvement of multiple regional lymph nodes; M1: gingival metastasis). The oral metastasis was radiologically described as an expansive lesion located in the mandibular left gingival vestibule, measuring approximately 30 mm in diameter.

The patient was therefore referred to the Oncology Department to initiate appropriate oncological therapy and underwent chemotherapy according to a Cisplatin–Etoposide–Durvalumab regimen administered over six cycles, once every 28 days.

At 3 month follow-up, a total body CT examination showed unchanged dimensions of the primary lung lesion and of the nodules.

The oral metastasis, after 7 months, showed a dimensional reduction from 30 mm to 11 mm and a reduction in the left level 1B lymph node from 13 mm to 7 mm. Clinically, no significant alterations of the alveolar mucosa were observed.

### 2.2. Systematic Review

A systematic review of the published cases of oral metastasis due to small cell lung cancer (SCLC) was performed to analyze the available literature, including our case report. This systematic review was conducted in accordance with the Preferred Reporting Items for Systematic Reviews and Meta-Analyses extension for systematic reviews (PRISMA2020) guidelines [17]. The inclusion criteria were full papers, English language, observational clinical studies (case reports/series) and prospective and retrospective (cohort and case–control studies), and randomized clinical trials, if available. Patients affected by oral metastasis due to SCLC were included in this review. Only cases with histopathological diagnosis of oral metastasis were included. The exclusion criteria were the lack of a histological diagnosis of oral metastasis in patients with SCLC and non-English studies. A comprehensive and systematic electronic search of Medline (via PubMed), Scopus, and the Cochrane Central Register of Controlled Trials was conducted from database inception. The final search was conducted in October 2025. Electronic searches were performed using a combination of the following Medical Subject Headings terms and free-text words: “Small Cell Lung Carcinoma”[Mesh] OR “small-cell lung cancer”[tiab] OR “small-cell lung carcinoma”[tiab] OR “oat cell carcinoma”[tiab] OR “oat cell lung cancer”[tiab]) AND “Mouth Neoplasms”[Mesh] OR “Neoplasm Metastasis”[Mesh] OR “oral metastasis”[tiab] OR “oral metastases”[tiab] OR gingiv*[tiab] OR tongue[tiab] OR mandible[tiab] OR maxilla[tiab] OR palate[tiab]. A manual search was conducted for articles published in the following journals: Oral Oncology; Clinical Oral Investigations; Journal of Oral Pathology and Medicine; Oral Surgery, Oral Medicine, Oral Pathology, and Oral Radiology; Head and Face Medicine; and Oral Diseases. Additionally, the bibliographies of all papers were checked to select other potentially relevant studies. The studies’ eligibility was independently assessed in a nonblinded and standardized manner by two reviewers (G.G. and I.C.). In the first round, records were screened by the titles and abstracts. In the second round, the full texts of the eligible papers were read. Only the articles that fulfilled the inclusion criteria were included in this systematic review. If there was disagreement regarding the selection of a paper, it was evaluated by a third reviewer (C.L.) for the final decision. Data were extracted using a predefined form using including author year, age, sex, primary tumor location, oral metastatic site, clinical presentation, radiological findings, histopathological and immunohistochemical features, treatment, follow up, and data when available. Missing data were not considered and thus not included in the analysis. Because all included studies were case reports, they were methodologically assessed using the Joanna Briggs Institute Appraisal Check-list for case reports.

## 3. Results

The review was conducted following the PRISMA 2020 checklist (Supplementary Materials).

During the article screening process, a total of 1519 articles were selected: 474 from PubMed, 802 from Scopus, and 243 from Web of Science. After excluding articles that did not meet the inclusion criteria and removing duplicates, 11 articles were identified for full-text assessment. Of the 11 selected articles, only 10 full texts were obtained, despite attempts to contact the corresponding author.

**Figure 7:**
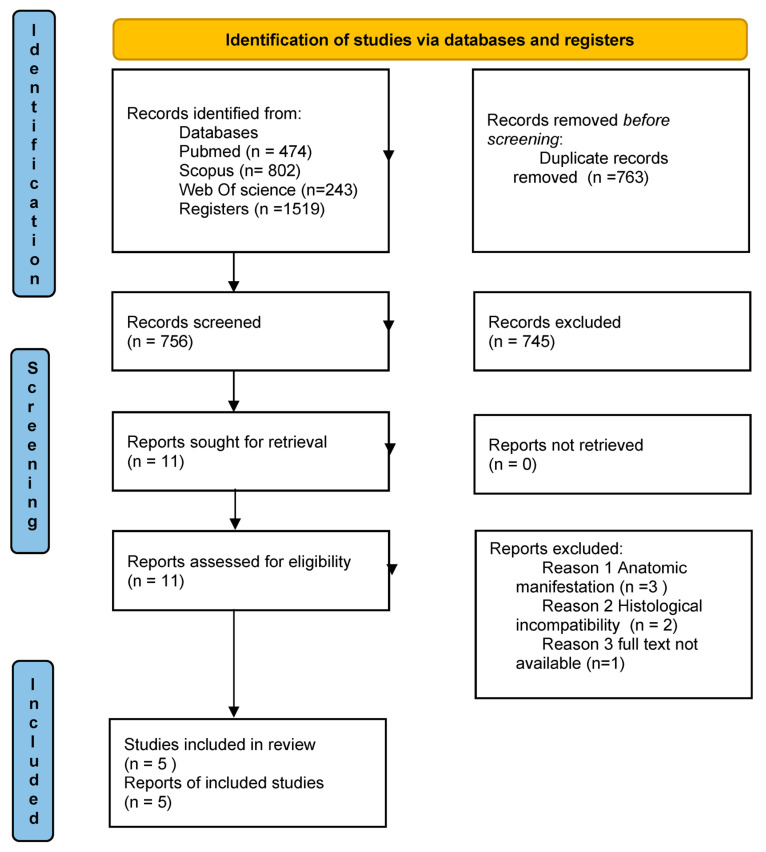
PRISMA 2020 flow diagram for new systematic reviews which included searches of databases and registers only.

Out of these 11 articles, after applying additional inclusion criteria, only five were deemed eligible for the review [18,19,20,21,22]. The data are presented according to a PRISMA flow diagram, including the reasons for article exclusion (Figure 7).

### Characteristics of the Included Studies and Summary of Results

In our review, only case reports were included. Thus, five oral metastases from small-cell neuroendocrine carcinoma originating from the lung were included (Table 2).

A total of six patients were identified with oral metastasis from small-cell neuroendocrine carcinoma [18,19,20,21,22], including this case report. Three patients were male [19,21,22] and three were female [20,22], with ages ranging from 37 to 69 years and a mean age of 58.4. The most common location was the mandible in five cases [18,19,20,21], while one case [22] was found in the maxilla. Specific characteristics of the included studies are reported in Table 2.

Radiographic imaging (i.e., orthopantomography and Dentascan) was reported in four cases [18,20,21], showing radiolucent lesions with erosive capability affecting the mandible. Clinically, the lesions ranged in size from 0.5 × 0.7 mm (bone lesion) to 5 × 5 mm, exhibiting an exophytic, soft, and tender appearance, erythematous coloration, rapid growth, and evidence of bone erosion.

The onset of oral metastases occurred after the diagnosis of the primary tumor [18,19,20,21,22]: some authors reported a 6-month time-lapse [20,22], others a 12-month time-lapse [21], while some authors did not provide data on the time elapsed since diagnosis of the primary tumor [18,19]. In this case, the oral metastasis represented the initial clinical presentation and, following histopathological evaluation, led to the diagnosis of a small-cell neuroendocrine carcinoma originating from the lung.

Histological examination was performed in all included articles and are reported in Table 3.

The articles included in the review reported different therapeutic approaches, such as chemotherapy and chemoradiotherapy. This case report and two articles reported chemotherapy as the sole treatment modality [19,21], while the remaining three articles [18,20,22] described combined chemoradiotherapy approaches.

In only one case [19], the surgical resection of the oral metastasis was proposed in order to improve quality of life.

The survival time reported in the included studies ranged from 3 months after diagnosis of the primary tumor [19] to more than 12 months [21], with survival periods of 5, 6, and 7 months reported in other cases [18,20,22]. The clinical case presented by us is currently under follow-up at 7 months. Only one study [21] reported complete healing of the oral metastasis, while the remaining studies did not provide information regarding the follow-up of oral metastasis [18,19,20,22]. The methodological quality of the included studies was heterogeneous. The majority of reports presented the correct amount of information but with limitations in different aspects, like incomplete timelines, lack of standardized follow-up and inconsistent reporting of adverse events. The rick of bias was considered moderate to high (Table 4).

## 4. Discussion

In the oral cavity, metastasis from lung tumors is very uncommon, accounting for a percentage between 11% and 16.6% [2,5]. Among these, the adenocarcinoma of the lung is the most commonly involved [3,16]. Nevertheless, few cases of metastatic lesions attributable to small-cell neuroendocrine carcinoma may also be found [16].

Small-cell neuroendocrine carcinomas (SCLC) account for approximately 10–15% of all lung tumors [11,23,24], showing a comparable incidence in both genders [25,26]. Despite advances in therapy, the prognosis of these patients remains poor, due to diagnosis occurring at advanced stages of the disease [22,26]. SCLC shows rapid growth, which allows extensive dissemination even in short periods of time [14,27]. The typical symptomatology often consists of cough, chest pain, weight loss, and fatigue; however, the onset of symptoms is frequently associated with an advanced stage of the disease [14,27]. The malignant cells of SCLC can disseminate to distant anatomical sites through various mechanisms, typically affecting structures and organs such as the brain, bones, lymph nodes, and liver [28,29]. This study identified only six cases of oral metastasis in the oral cavity [18,19,20,21,22].

These metastatic lesions are often difficult to diagnose due to their variable characteristics, such as a polypoid or exophytic appearance, highly vascularized and/or edematous [18,19,20,21,22], showing a close resemblance to some benign hyperplastic or reactive oral lesions (e.g., fibromas, epulides, pyogenic granulomas, or peripheral giant cell granulomas) [2,16].

Despite the apparently benign appearance, the symptomatology was characterized by intense pain on palpation and reduced mobility, which are typical features of malignant lesions (e.g., oral squamous cell carcinoma). Histopathological analysis remains the gold standard in the diagnosis of these lesions due to their highly variable clinical aspects [2,16].

The mandible is more commonly affected by oral metastases compared to the maxilla [19,20,21]. In agreement with the data found in our review, only one case [22] reports the location of the metastatic lesion in the hard palate.

Among the oral cavity, the attached gingiva is the most frequently affected tissue [2,18,30,31], followed by the epithelial lining of the tongue [1,2,18,22,31] and the keratinized mucosa of the palate [18,22]. The attached gingiva contains a dense capillary plexus; the presence of chronic inflammation due to periodontal disease increases its permeability, favoring the passage of metastatic cells from the bloodstream to neighboring tissues [2]. Tooth extractions may be indirectly involved, promoting the formation of a clot rich in inflammatory cells (IL-1, TNF-α) [2], providing a favorable “microenvironment” for the engraftment of metastatic cells [16,32].

The articles included in our review show an altered periodontal status in patients with metastases [20,29]; only one article [21] reported the onset of the metastatic lesion following a tooth extraction [21]. In our case, the patient had an altered periodontal condition; other authors did not specify the oral health status of the patient [18,22].

Radiographically, metastatic lesions present a radiolucent, clearly erosive appearance, with poorly defined margins [5]. In our review, some authors [19,21,22] report the typical erosive and irregular appearance associated with malignant lesions, while one article reports a cyst-like appearance, with well-defined margins but very evident symptomatology [21].

In the presented case, immunohistochemistry allowed the identification of neuroendocrine and pulmonary origin (TTF-1, CD56, Synaptophysin, and Chromogranin A), in accordance with WHO criteria for high-grade neuroendocrine tumors [11]. Other articles included in the review [31,33,34] report compatibility with the markers TTF-1, Synaptophysin, and Chromogranin A. A comparison between the main markers is reported in Table 1. One article performed a fine-needle aspiration biopsy, providing only cytological data for the diagnosis [32], and another case did not report histological data other than the diagnosis [17]. Our case is the only one showing positivity for the marker CD56 (Neural Cell Adhesion Molecule).

The proposed treatments for metastatic small-cell neuroendocrine carcinoma are varied, based on chemotherapy with or without radiotherapy. Surgical resection of oral metastases is considered only when pain negatively affects the patient’s quality of life [31].

In our review, different chemotherapeutic drugs were used, combined or not with radiotherapy [18,19,20,21,22]. Several authors [19,20,22] propose an approach based on cytotoxic drugs such as Cisplatin [20] or Carboplatin [19] in combination with Etoposide. Our patient underwent a pharmacological regimen based on Cisplatin and Etoposide for six cycles every 28 days. Some studies report that the use of these two cytotoxic drugs together with radiotherapy (45 Gy once or twice daily) may increase the 5-year survival rate up to 23–44% [35]. One article reports on the use of Paclitaxel 60 mg/m^2^ [21], a drug used in cases of very advanced, locally refractory disease, in comparison to conventional treatments [33].

One study reports the use of radiotherapy in combination with chemotherapy [20], with a cumulative radiation dose of 5000 Gy. Some authors [34] report that combined treatment, in advanced oncological situations, increases the survival rate from 3% to 13% in the first two years. Another article [18] reports radiotherapy as the only treatment. In our case, radiotherapy was not considered appropriate.

The prognosis of SCLC is poor, due to the absence of symptoms until an advanced stage is reached and due to early metastasis [19]. A study by Baugh et al. showed an average survival rate of 19 months in patients undergoing chemotherapy, compared with 11 months in those who did not receive chemotherapy for tumors involving the head and neck region [32]. Various studies report a survival rate ranging from two weeks to more than four years [33,34,35,36,37,38,39,40,41].

## 5. Conclusions

Although metastases from small-cell neuroendocrine carcinoma originating from the lung are rare, they should be considered in the differential diagnosis of benign lesions. Lesions with rapid growth and changes in shape or color should raise suspicion of malignancy. Following the clinical examination, histological and immunohistochemical evaluation are necessary for a correct diagnosis. The life expectancy of these patients remains unfavorable, due to the late diagnosis of the tumor.

## Figures and Tables

**Table 1:** Immunohistochemical markers evaluated in the present case.

Marker	Result	Diagnostic Interpretation
AE1/AE3	Positive	Supports epithelial differentiation
INSM1	Positive	Supports neuroendocrine differentiation
Synaptophysin	Positive	Supports neuroendocrine differentiation
Chromogranin A	Positive	Supports neuroendocrine differentiation
CD56	Positive	Supports neuroendocrine differentiation
TTF-1	Positive	Supports pulmonary origin in the appropriate clinical and radiological context
Ki-67	Approximately 90%	High proliferative index, consistent with high-grade neuroendocrine carcinoma
INI-1	Retained	Retained nuclear expression
P40	Negative	Does not support squamous differentiation
P63	Negative	Does not support squamous differentiation
IDHI	Negative	No supportive evidence of IDHI mutated lesion
NUT	Negative	Does not support NUT carcinoma
EBV/EBER	Negative	No evidence of EBV-associated lesion

**Table 2:** Summary of the reports of the included studies. NR: Not Reported.

Article	Age	Sex	Tumor Localization	Metastasis Site
Kim et al. 1979 [18]	60	Male	NR	Anterior Tongue
Pektas et al. 2013 [20]	50	Female	Left Ilary Region	Keratinized Gingiva (left mandible, second premolar)
Kalaitsidou et al. 2015 [19]	69	Male	Right Upper-Middle Lobe	Anterior Mandible
Thottian et al. 2017 [22]	38	Female	Peri-hilar Region	Left Posterior Hard Palate
Moraes et al. 2017 [21]	66	Male	NR	Keratinized Gingiva (left mandible, first premolar)
Gioco et al. 2026 (Current Study)	62	Female	Right Lobe	Keratinized Gingiva (left mandible)

**Table 3:** Histopathology and therapy of the included cases. NR: Not Reported, NS: Not Specified.

Article	Histopathology	Therapy	Metastasis Size (cm)	Follow-Up (Months)
Kim et al. 1979 [18]	NR	Chemotherapy (NS) Radiotherapy (30 Gy)	NR	6
Pektas et al. 2013 [20]	FNAC; fused cells; visible mitoses	Carboplatin; Etoposide	4 × 3	7
Kalaitsidou et al. 2015 [19]	LMW-keratin+; CK7; TTF-1; Chromogranin A; Synaptophysin	Carboplatin; Etoposide	4 × 5	3
Thottian et al. 2017 [22]	Synaptophysin; Chromogranin A; Cytokeratin; Ki-67/MIB-1 > 90%	Cisplatin; Etoposide; Radiotherapy (45 Gy)	4 × 2	5
Moraes et al. 2017 [21]	CK7; Chromogranin A; TTF-1	Paclitaxel 60 mg/m^2^	4 × 5	12
Gioco et al. 2026 (Current Study)	AE1/AE3; INSM1; Synaptophysin; Chromogranin A; CD56; TTF-1	Cisplatin; Etoposide; Durvalumab	2 × 3	>7

**Table 4:** Summary of JBI critical appraisal of the included case reports.

Study	Demographics	Time-Line	Clinical Condition	Diagnosis	Treatment	Follow-Up	Adverse Events	Overall Quality
Kim et al. 1979 [18]	Yes	Unclean	Yes	Unclear	Yes	Partial	No	Include with caution
Pektas et al. 2013 [20]	Yes	Partial	Yes	Partial	Yes	Yes	No	Included with caution
Kalaitsidou et al. 2015 [19]	Yes	Yes	Yes	Yes	Yes	Yes	No	Include
Thottian et al. 2017 [22]	Yes	Yes	Yes	Yes	Yes	Partial	No	Include
Moraes 2017 [21]	Yes	Yes	Yes	Yes	Yes	Yes	No	Include
Gioco et al. 2026 (Current Study)	Yes	Yes	Yes	Yes	Yes	Yes	No	Included

## Data Availability

The authors confirm that the data supporting the findings of this study are available within the article.

## Supplementary Materials

The following supporting information can be downloaded at: https://www.mdpi.com/article/10.3390/jcm15103772/s1, PRISMA 2020 Checklist.

## References

[1] Hirshberg A., Buchner A. (1995). Metastatic tumours to the oral region: An overview. Eur. J. Cancer B Oral Oncol..

[2] Hirshberg A., Shnaiderman-Shapiro A., Kaplan I., Berger R. (2008). Metastatic tumours to the oral cavity: Pathogenesis and analysis of 673 cases. Oral Oncol..

[3] Orlandi A., Basso M., Di Salvatore M., Federico F., Cassano A., Barone C. (2011). Lung adenocarcinoma presenting as a solitary gingival metastasis: A case report. J. Med. Case Rep..

[4] Murillo J., Bagan J.V., Hens E., Diaz J.M., Leopoldo M. (2013). Tumors metastasizing to the oral cavity: A study of 16 cases. J. Oral Maxillofac. Surg..

[5] Tan Q., Xu J., Xu W., Lu H. (2024). Surgery and superficial x-ray radiotherapy for keloids of the preauricular and contralateral ear lobe: Case report. Clin. Case Rep..

[6] Scilla F., Rupe C., Gioco G., Raffaelli L., Lococo F., Mazzarella C., Rindi G., Patini R., Lajolo C. (2025). Rapid growing mass of the mandible due to an oral metastasis of thymoma: Case report of an extremely rare localization and review of published cases. Heliyon.

[7] Gioco G., Patini R., Marchesini D., Rupe C., Staderini E., Lucchese A., De Corso E., Lajolo C. (2025). Oral metastasis from colorectal adenocarcinoma: Report of a new case and a scoping review. Case Rep. Dent..

[8] Sung H., Ferlay J., Siegel R.L., Laversanne M., Soerjomataram I., Jemal A., Bray F. (2021). Global cancer statistics 2020: GLOBOCAN estimates of incidence and mortality worldwide for 36 cancers in 185 countries. CA Cancer J. Clin..

[9] World Cancer Research Fund International Lung Cancer Statistics. Updated March 2022. https://www.wcrf.org/cancer-trends/lung-cancer-statistics.

[10] Schaal C., Chellappan S.P. (2014). Nicotine-mediated cell proliferation and tumor progression in smoking-related cancers. Mol. Cancer Res..

[11] Nicholson A.G., Tsao M.S., Beasley M.B., Borczuk A.C., Brambilla E., Cooper W.A., Dacic S., Jain D., Kerr K.M., Lantuejoul S. (2022). The 2021 WHO classification of lung tumors: Impact of advances since 2015. J. Thorac. Oncol..

[12] Kocher F., Hilbe W., Seeber A., Pircher A., Schmid T., Greil R., Auberger J., Nevinny-Stickel M., Sterlacci W., Tzankov A. (2015). Longitudinal analysis of 2293 NSCLC patients: A comprehensive study from the TYROL registry. Lung Cancer.

[13] Dingemans A.-M., Früh M., Ardizzoni A., Besse B., Faivre-Finn C., Hendriks L., Lantuejoul S., Peters S., Reguart N., Rudin C. (2021). Small-cell lung cancer: ESMO clinical practice guidelines for diagnosis, treatment and follow-up. Ann. Oncol..

[14] Caballero Vazquez A., Garcia Flores P., Romero Ortiz A., Garcia del Moral R., Alcázar-Navarrete B. (2020). Small cell lung cancer: Recent changes in clinical presentation and prognosis. Clin. Respir. J..

[15] Farago A.F., Keane F.K. (2018). Current standards for clinical management of small cell lung cancer. Transl. Lung Cancer Res..

[16] Gupta S., Jawanda M.K., Kedia N.B., Deb A.R., Ganganna A., Saurabh K., Yadav S.K., Yadav A.B. (2022). Lung cancer metastasis to oral soft tissues; Systematic review of 122 cases. J. Clin. Exp. Dent..

[17] Page M.J., McKenzie J.E., Bossuyt P.M., Boutron I., Hoffmann T.C., Mulrow C.D., Shamseer L., Tetzlaff J.M., Akl E.A., Brennan S.E. (2021). The PRISMA 2020 statement: An updated guideline for reporting systematic reviews. BMJ.

[18] Kim R.Y., Perry S.R., Levy D.S. (1979). Metastatic carcinoma to the tongue: A report of two cases and a review of the literature. Cancer.

[19] Kalaitsidou I.G., Astreidis I.T., Kontos K.I., Lazaridou M.N., Bourlidou E.T., Gerasimidou D.K., Vladika N.P., Mangoudi D.L. (2015). Metastatic tumours to the oral cavity: Report of three cases. J. Oral Maxillofac. Res..

[20] Pektas Z.O., Gunhan O. (2013). Cytologically diagnosed metastatic small cell lung carcinoma in the mandibular soft tissue. Saudi Med. J..

[21] Moraes R.M., Alves F.A., Carvalho B.F.C., Costa F.D.A., Lopes R.N., Jaguar G.C. (2017). Mandible metastasis of small cell lung cancer mimicking a residual cyst. Autops. Case Rep..

[22] Thottian A.G.F., Pathy S., Gandhi A.K., Malik P., Nambirajan A. (2017). Coughing up—Small cell carcinoma lung with gingival metastasis. J. Egypt. Natl. Cancer Inst..

[23] Rudin C.M., Brambilla E., Faivre-Finn C., Sage J. (2021). Small-cell lung cancer. Nat. Rev. Dis. Prim..

[24] Pandjarova I., Mercieca D., Gijtenbeek R.G.P., Pereira J.O., Fantin A., Castaldo N., Keramida E., Pannu K., Konsoulova A., Aujayeb A. (2024). Small cell lung cancer and neuroendocrine tumours. Breathe.

[25] Franco F., Carcereny E., Guirado M., Ortega A.L., López-Castro R., Rodríguez-Abreu D., García-Campelo R., del Barco E., Juan O., Aparisi F. (2021). Epidemiology, treatment, and survival in small cell lung cancer in Spain: Data from the Thoracic Tumor Registry. PLoS ONE.

[26] National Comprehensive Cancer Network NCCN Clinical Practice Guidelines in Oncology (NCCN Guidelines) for Small Cell Lung Cancer V.2.2024. http://www.nccn.org/professionals/physician_gls/PDF/occult.pdf.

[27] Soomro Z., Youssef M., Yust-Katz S., Jalali A., Patel A.J., Mandel J. (2020). Paraneoplastic syndromes in small cell lung cancer. J. Thorac. Dis..

[28] Tamura T., Kurishima K., Nakazawa K., Kagohashi K., Ishikawa H., Satoh H. (2015). Hizawa, Specific organ metastases and survival in metastatic non-small-cell lung cancer. Mol. Clin. Oncol..

[29] Zhou Q., Zu L., Li L., Chen X., Chen X., Li Y., Liu H., Sun Z. (2014). Screening and establishment of human lung cancer cell lines with organ-specific metastasis potential. Chin. J. Lung Cancer.

[30] Hirshberg A., Berger R., Allon I., Kaplan I. (2014). Metastatic tumours to the jaws and mouth. Head Neck Pathol..

[31] Hirshberg A., Leibovich P., Buchner A. (1993). Metastatic tumors to the oral mucosa: Analysis of 157 cases. J. Oral Pathol. Med..

[32] Allon I., Pessing A., Kaplan I., Allon D.M., Hirshberg A. (2014). Metastatic tumors to the gingiva and the presence of teeth as a contributing factor: A literature analysis. J. Periodontol..

[33] von Eiff D., Bozorgmehr F., Chung I., Bernhardt D., Rieken S., Liersch S., Muley T., Kobinger S., Thomas M., Christopoulos P. (2020). Paclitaxel for treatment of advanced small cell lung cancer (SCLC): A retrospective study of 185 patients. J. Thorac. Dis..

[34] Levy A., Botticella A., Le Péchoux C., Faivre-Finn C. (2021). Thoracic radiotherapy in small cell lung cancer—A narrative review. Transl. Lung Cancer Res..

[35] Turrisi A.T., Kim K., Blum R., Sause W.T., Livingston R.B., Komaki R., Wagner H., Aisner S., Johnson D.H. (1999). Twice-daily compared with once-daily thoracic radiotherapy in limited small-cell lung cancer treated concurrently with cisplatin and etoposide. N. Engl. J. Med..

[36] Baugh R.F., Wolf G.T., Krause C.J., Beals T.F., Forastiere A. (1986). Small cell carcinoma of larynx: Results of therapy. Laryngoscope.

[37] Curien R., Moizan H., Gerard E. (2007). Gingival metastasis of a bronchogenic adenocarcinoma: Report of a case. Oral Surg. Oral Med. Oral Pathol. Oral Radiol. Endod..

[38] Sauerborn D., Vidakovic B., Baranovic M., Mahovne I., Danic P., Danic D. (2011). Gastric adenocarcinoma metastases to the alveolar mucosa of the mandible: A case report and review of the literature. J. Craniomaxillofac. Surg..

[39] Jain S., Kadian M., Khandelwal R., Agarwal U., Bhowmik K.T. (2013). Buccal metastasis in a case of carcinoma breast: A rare case report with review of literature. Int. J. Surg. Case Rep..

[40] Zachariades N. (1989). Neoplasms metastatic to the mouth, jaws and surrounding tissues. J. CranioMaxillofac. Surg..

[41] Irani S., Moshref M., Lotfi A. (2004). Metastasis of a gastric adenocarcinoma to the mandible. Oral Oncol. Extra.

